# Supplementary material for: Phenotype and function of IL-10–producing NK cells in individuals with malaria experience
Source: JCI Insight. 2025 May 8;10(9):e183076. doi: 10.1172/jci.insight.183076 (PMC12128992; doi:10.1172/jci.insight.183076)
Supplement: Supplemental data [file jciinsight-10-183076-s267.pdf]

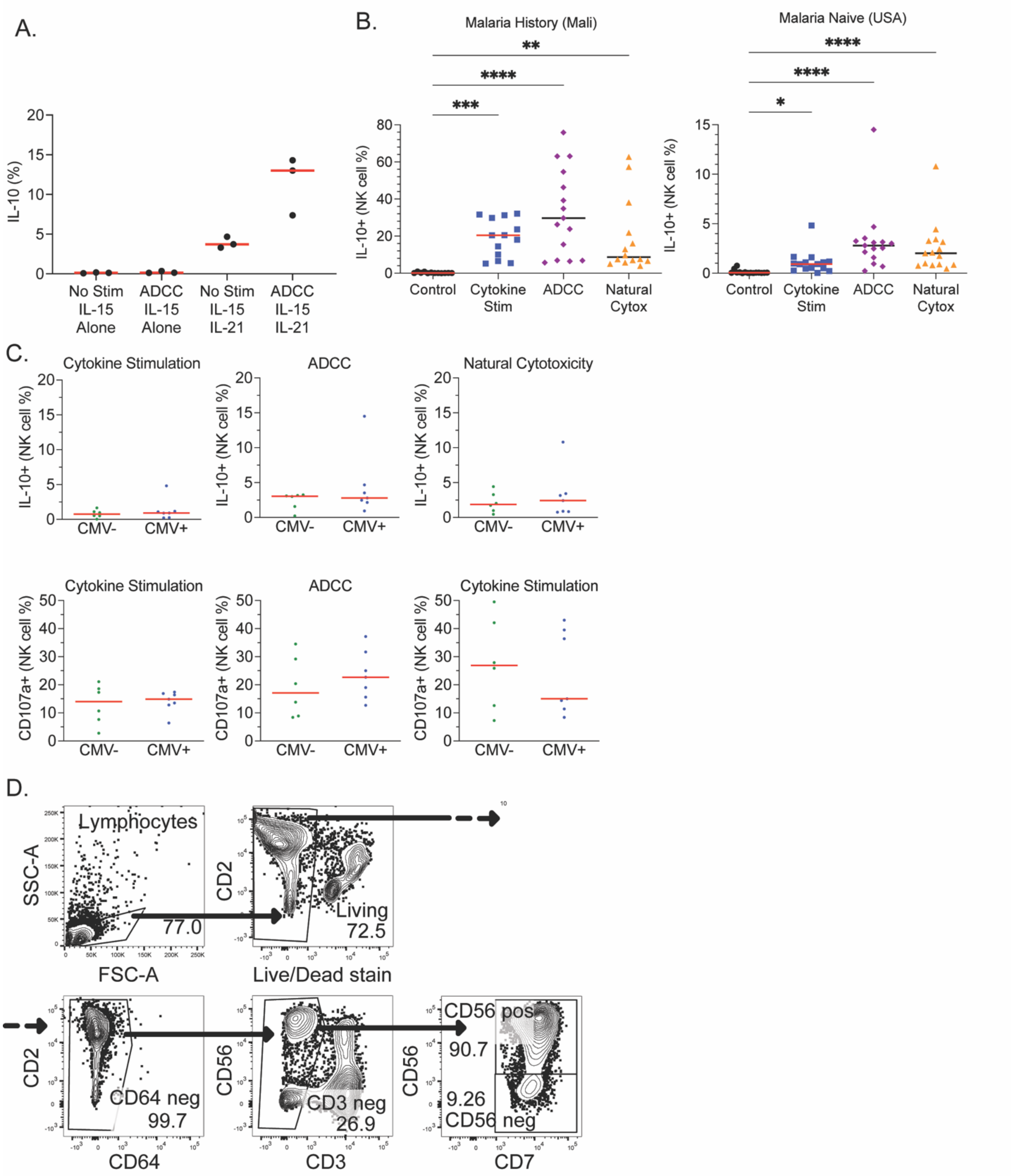

Supplemental Figure 1. Comparison of stimulation conditions, CMV seropositivity, and example Flow Cytometry gating. (A) IL-10 expression after incubation for 6 days with IL-15 alone or IL-15 with IL-21 without or with ADCC stimulation on Day 6. (B) The same data shown in Figure 1 and Figure 2 was combined into one graph to show the relationship between cellular assays. All combinations within each graph were tested for significance first with a Kruskal-Wallis test, then with a Dunn's multiple comparisons post-hoc test between groups. (**$p<0.01$, ***$p<0.001$, ****$p<0.0001$) (C) IL-10 (top row)

and CD107a (bottom row) expression in CMV seropositive and seronegative individuals (USA). Groups were compared using Wilcoxon signed-rank tests (*p<0.05, **p<0.01, ***p<0.001); orange bars represent median values. (D) Example gating of a representative malaria experienced subject's cells via flow cytometry.

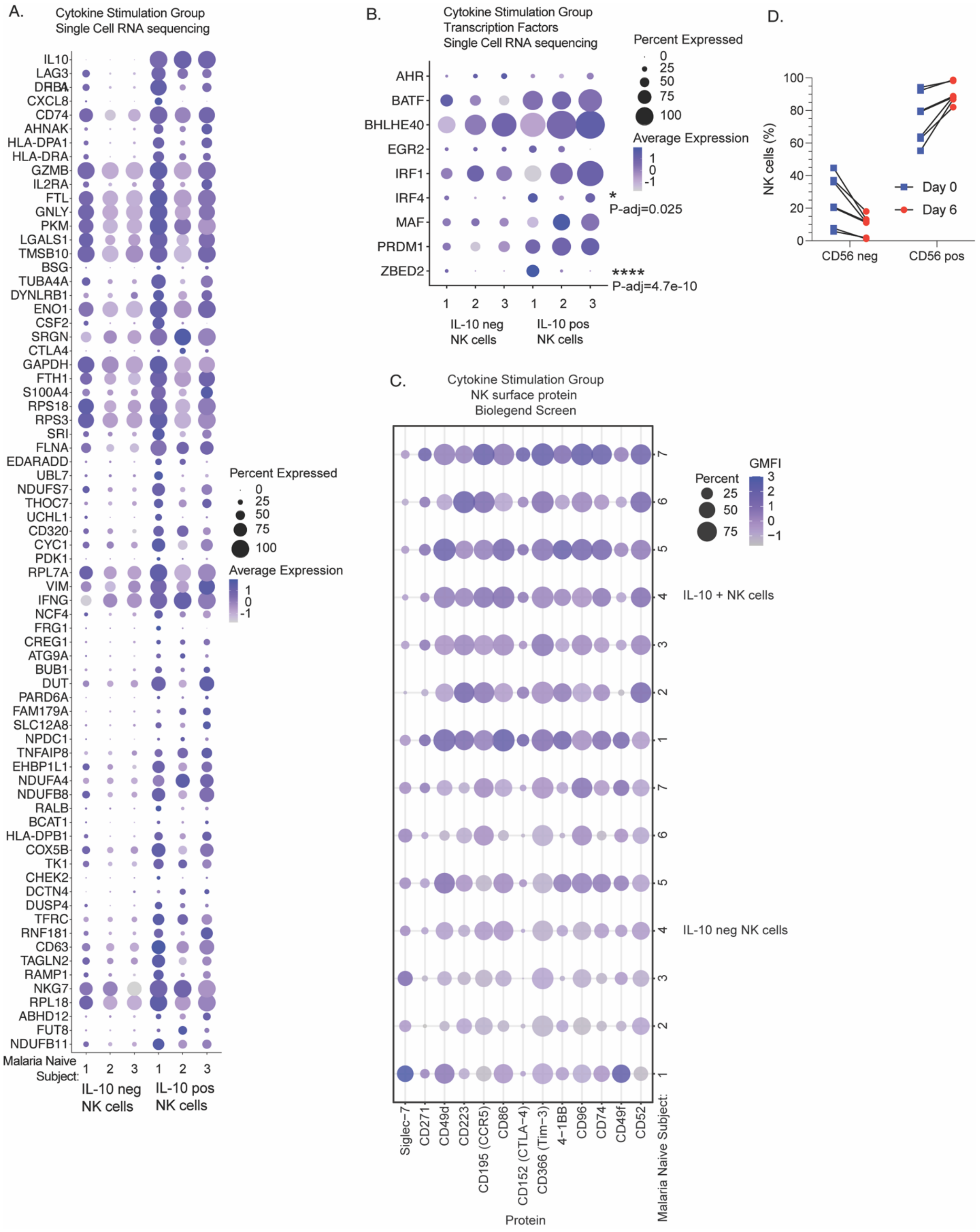

Supplemental Figure 2. Initial single cell RNA sequencing and surface protein screen to identify potential additional markers associated with IL-10 producing NK cells. (A) Single-cell RNA sequencing was done on three malaria-naïve individuals NK cells that had been through the Cytokine

stimulation protocol. In the identified NK cells, IL-10 negative and IL-10 positive NK cells were compared, and the top significant candidate markers are shown. Circle size indicates percent of NK cells positive for the gene. Color intensity indicates expression level up or down. (B) Single-cell RNA sequencing comparing known Tr1 CD4 T cell transcription factors between IL-10 negative and IL-10 positive NK cells post-cytokine stimulation. Circle size indicates percent of NK cells positive for the gene. Color intensity indicates expression level up or down. Significant genes highlighted with asterisks and p-values on the right side. (C) Using a the Biolegend LEGENDScreen surface receptor screen, six malaria-naïve individuals NK cells that had been through the Cytokine stimulation protocol were test for surface expression of 350 proteins. IL-10 negative and IL-10 positive NK cells were compared, and significant candidate markers are shown. Circle size indicates percent of NK cells positive for the gene. Color intensity indicates expression level up or down. Data was analyzed using students t-test with Benjamini-Hochberg adjusted p-values. (D) Proportion of CD56 positive and CD56 neg that are Living, CD64-, CD3-, CD7+ at Day 0 and Day 6 post cytokine incubation.

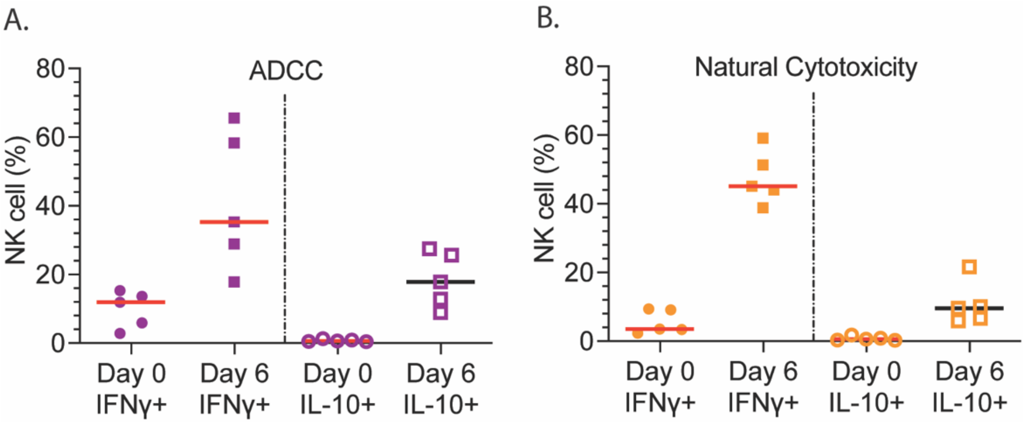

Supplemental Figure 3. NK cell production of IFNγ or IL-10 directly *ex-vivo* (Day 0) or 6 days after stimulation. (A-B) Malaria-experienced NK cells producing either IFNγ (Left) or IL-10 (Right) on Day 0 or 6 during ADCC (A) or Natural Cytotoxicity (B).

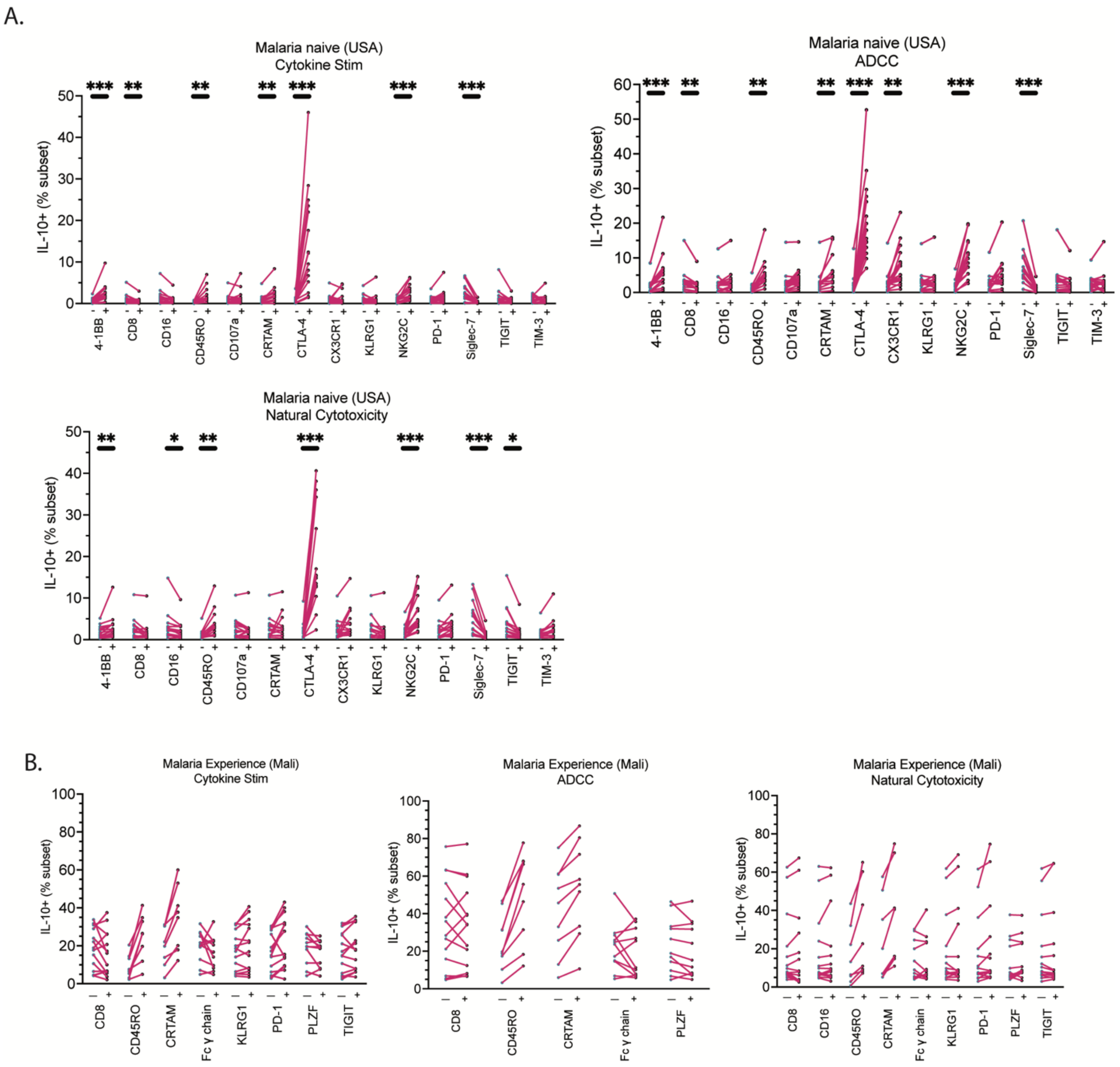

Supplemental Figure 4. IL-10 production delineated by NK cell marker for malaria- naïve (USA) and malaria-experienced (Mali) individuals. (A-B) IL-10 production by NK cells for Cytokine stimulation, ADCC, and Natural Cytotoxicity. Malaria-naïve (A) and malaria-experienced (B) IL-10 production by NK cells. Mann-Whitney non-parametric paired test done initially followed by a Bonferroni correction (14) to get the final p-value. * = p-value <0.05, ** = p-value <0.01, *** = p-value <0.001, **** = p-value <0.0001 post Bonferroni correction.

Supplemental Table 1. Demographic Characteristics of Malaria-experienced and Malaria-naïve Study Participants. For malaria-experienced participants (Mali, Africa), *n* = 22. For malaria-naïve individuals (Minnesota, USA), *n* = 22. Malaria-experienced participants were on average 15.5 years old (*SD* = 13.6).

[a] Reflects the number of malaria-experienced individuals who suffered at least 1 episode of malaria during the year blood samples were collected.

	Mali	*USA*
Gender		
Female	7	7
Male	15	15
Age (years)		
3 – 7	11	
8 – 16	4	
17 – 42	7	
Malaria episode during year of collection? [a]		
Yes	10	0
No	12	23

Supplemental Table 2. Reagents.

Reagent	Source	Identifier
**Biological Assay Reagents**		
Healthy adult whole blood	Memorial Blood Bank	N/A
RPMI-1640	Kd Medical	50-101-8907
Percoll	Millipore	GE17-0891-09
Ficoll	MP Biomedicals	ICN50494
ACK Lysis Buffer	Lonza	BP10-548E
X-VIVO 15 Serum Free Cell Medium	VWR	12001-988
PBS 10X	Corning	20-031-CV
Propiduim Iodide	Thermo Fisher	P1304MP
Heat-inactivated FBS	PEAK Serum	PS-FB1
Heat-Inactivated human male AB serum	PEAK Serum	PS-HS
DNAse	Roche	4716728001
EDTA	Thermo Fisher	BP2482-1
Pen/Strep	Gibco	1514022
Genatmicin	Sigma-Aldrich	G1272
BFA	Millipore Sigma	20350-15-6
Monensin	Millipore Sigma	M5273
Formaldehyde	Thermo Fisher	28908
Triton X-100	Thermo Fisher	BP151-500
BSA	MP Biomedicals	810681
Dimethyl sulfoxide	Sigma-Aldrich	D260-100ML
K562 WT Cell line	ATCC	CCL-243
anti-red blood cell rabbit polyclonal	Rockland	1094139
IL-12	R&D Systems	AF-219-NA
IL-15	NCI	N/A
IL-21	Biolegend	571204
**Critical Commercial Assays**		
LEGENDScreen Human PE Kit	Biolegend	7000007
IL-10 Secretion Assay Detection Kit (APC)	Miltenyi	130-090-761
**Software and Algorithms**		
FlowJo (10.8.1)	FlowJo	N/A
Excel (16.54)	Microsoft	N/A
RStudio (1.4.1717)	N/A	N/A
Prism (10)	GraphPad	N/A
Illustrator (25.4.1)	Adobe	N/A
**Other**		
Mr. Frosty	Thermo Fisher	5100-0001
RBC Purification Filter	Fenwal Inc.	RS-2000

Antibodies

Antigen	Clone	Fluorophore	Dilution	Manufacturer	Catalogue number
		**Intracellular**			
FcRγ	polyclonal	FITC	1 to 50	Millipore	FCABS400F
IFNγ	B27	PE-CF594	1 to 50	BD Horizon	562392
PLZF	Mags.21F7	PE	1 to 100	eBioscience	12-9320-82
		**Extracellular**			
4-1BB	4B4-1	BV711	1 to 50	Biolegend	311503
CD107a	H4A3	BV421	1 to 100	Biolegend	328626
CD14	63D3	BV510	1 to 100	Biolegend	367124
CD16	3G8	BUV496	1 to 100	BD Optibuild	612944
CD2	RPA-2.10	BUV395	1 to 100	Biolegend	563819
CD223 (LAG-3)	11C3C65	PerCP-Cy5.5	1 to 50	Biolegend	369312
CD3	SK7	BUV805	1 to 100	BD Biosciences	612893
CD355 (CRTAM)	Cr24.1	PerCP-Cy5.5	1 to 25	Biolegend	339112
CD45RO	UCHL1	BV785	1 to 50	Biolegend	304234
CD56	NCAM16.2	BUV563	1 to 100	BD Biosciences	612928
CD64	10.1	BUV737	1 to 75	BD Biosciences	612777
CD7	4H9	BV510	1 to 25	BD Biosciences	568325
CD8a	SK1	BV510	1 to 50	Biolegend	344732
CTLA-4	BNI3	R718	1 to 25	Biolegend	369614
CX3CR1	2A9-1	PE-CF594	1 to 100	Biolegend	341624
IL-10	N/A	APC	1 to 10	Miltenyi	130-090-761
KLRG1	SA231A2	KIRAVIA Blue520	1 to 50	Biolegend	367726
NKG2c	REA205	PE-Vio 770	1 to 50	Miltenyi	130-120-449
PD-1	PD1.3.1.3	PE	1 to 50	Miltenyi	130-117-384
Siglec-7	REA214	APC-Vio770	1 to 25	Miltenyi	130-101-009
TIGIT	A15153G	BV605	1 to 25	Biolegend	372712
Tim-3 (CD366)	F38-2E2	BV650	1 to 50	Biolegend	345028
Viability Dye		UV Blue (UV 450)	1 to 300	Tonbo	13-0868-T100
